# Conservative treatment for equinus deformity in children with cerebral palsy using an adjustable splint-assisted ankle-foot orthosis

**DOI:** 10.1097/MD.0000000000008186

**Published:** 2017-10-27

**Authors:** Wei Chen, Xiaoyu Liu, Fang Pu, Yang Yang, Lizhen Wang, Hong Liu, Yubo Fan

**Affiliations:** a Key Laboratory of Rehabilitation Technical Aids, Ministry of Civil Affair, School of Biological Science and Medical Engineering, Beihang University; b State Key Laboratory of Virtual Reality Technology and Systems, Beihang University; c National Research Center for Rehabilitation Technical Aids; d Rokab Pedorthic Center, Beijing, P.R. China.

**Keywords:** adjustable splint, ankle-foot orthosis, cerebral palsy, equinus

## Abstract

**Background:**

A novel splint, the assisting ankle-foot orthoses (AFO), was developed to provide adjustable sustained stretching to improve conservative treatment for equinus deformities in children with cerebral palsy (CP). The treatment effect was validated by follow-up visits.

**Methods:**

This study involved subjects between 2 and 12 years old, including 28 CP children treated with splint-assisted AFO correction, 30 CP children treated with static AFO correction, and 30 normal children with typical development (TD). Quantitative pedobarographic measurements were taken to evaluate the effect of splint-assisted AFO correction. The heel/forefoot ratio was introduced to indicate the degree of the equinus deformity during treatment.

**Results:**

The results showed that the heel/forefoot ratios were 1.41 ± 0.26 for the TD children; 0.65 ± 0.41, 1.02 ± 0.44, and 1.24 ± 0.51 for the splint-assisted AFO correction before and after 6-month and 12-month treatments; 0.59 ± 0.37, 0.67 ± 0.44, and 0.66 ± 0.42 for the static AFO correction before and after 6-month and 12-month treatments.

**Conclusions:**

This study suggests that correction with the adjustable splint-assisted AFO is an effective treatment for equinus deformity in CP Children.

## Abstract 2

Supplemental Digital Content is available in the text

## 1 Introduction

Spastic equinus is the most common movement disorder in children with cerebral palsy (CP).^[1]^ Equinus deformities result from tight calf muscles or Achilles tendons, causing a shift of the force bearing point from the hindfoot to the forefoot. Since children's feet are relatively flexible and adaptable, nonoperative management is typically used a first course of treatment.^[2–4]^ Passive stretching is an important nonoperative treatment for equinus correction,^[5]^ used in the belief that tight muscles and tendons would be relaxed and lengthened.^[6]^ Strong evidence indicates that sustained stretching can significantly improve the outcome of correction for CP children.^[7,8]^

Ankle-foot orthoses (AFO), which serve to limit ankle plantar flexion and provide passive stretching for the tight soft tissues, are considered to be an effective conservative treatment for preventing the progression of equinus deformities.^[2,9–12]^ However, the long-term clinical effectiveness of using an AFO is still unclear.^[10,13]^ Most AFO maintain the ankle in a neutral position, but do not provide methods for adjustable stretching of the tight muscles or tendons. During the treatment period, CP children would exhibit various degrees of equinus deformity. Considering this variation, for optimum treatment, the soft tissues should be subjected to different stretching strategies; for example, varying the duration and magnitude of force.^[14,15]^

In this study, an adjustable splint was developed to provide changeable sustained stretching to tight muscles and tendons in CP children. By adjusting the ankle dorsiflexion angle, tight muscles and tendons of the affected foot could be subjected to effective, customized stretching over the course of treatment. To evaluate this novel corrective technique, pedobarographic analysis was used to compare the outcome of long-term treatment using AFO + stretching and static AFO correction.

## 2 Method

### 2.1 Participants

Eighty children with CP who were scheduled for equinus correction were recruited from the Rokab Pedorthic Center in Beijing between 2012 and 2014. The children were between 2 and 12 years old and displayed either diplegic or hemiplegic CP with true equinus^[16]^ (16 aged 2–4, 18 aged 4–6, 16 aged 6–8, 16 aged 8–10, and 14 aged 10–12). The inclusion criteria were as follows: subjects with CP having spasticity below grade IV, as measured by the modified Ashworth scale (MAS)^[17,18]^; no previous surgery on the foot or ankle; no calf tightness; and during the test, the subjects were required to stand unaided without losing their balance^[19]^ and could be maintained temporarily for at least 5 seconds. These CP children were randomly assigned to either receive or not receive stretching treatment according to an odd-even principle from each age group according to the previous studies.^[20–23]^ For CP children in the same age group, an odd visiting sequence was used for the AFO + stretching group and an even visiting sequence as assigned to the static AFO group. Usual physical therapy was required. Thirty children who showed typical development (TD) were also recruited prospectively as a comparison group. Demographic characteristics were collected for the 3 study groups (AFO + stretching, static AFO, and TD). Informed written consents were obtained from the parents of each subject, in accordance with clinical protocols. This study was approved by the Science and Ethics Committee of the School of Biological Science and Medical Engineering in Beihang University, Beijing, China, on February 5, 2012 (Approval ID: 20120205).

### 2.2 Procedure

**Figure 1:**
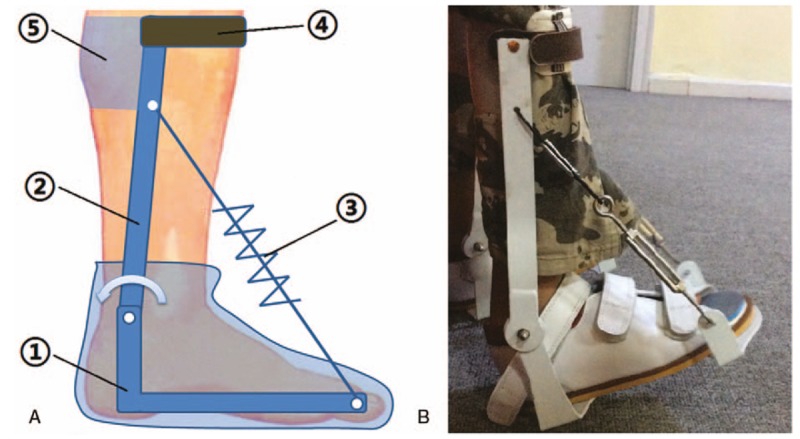
(A) Schematic of the adjustable dynamic splint which consists of (1) foot plate, (2) support bar, (3) adjustable screw bar, (4) strap holder and (5) shank polypropylene holder. (B) Splint being used to stretch the gastrocnemius/soleus muscle and Achilles tendon of a spastic CP child.

A customized adjustable splint was developed for stretching the muscles and Achilles tendon. The splint is intended for daily use, so was designed to be comfortable to wear and not to cause any unnecessary distress or discomfort to the user. Figure 1 shows the mechanism of this adjustable splint. By adjusting the screw bars, the angle of ankle dorsiflexion could be changed, subjecting the muscles to passive stretching. The splint was set on the patient's foot and the support bars were fixed on the shank. Then, the two screw bars were gradually shortened to increase the ankle dorsiflexion angle. When the patient's pain threshold was reached (verbally indicated), the adjustment was stopped and the screw bars were fixed in place. Each patient was instructed to perform muscle stretching using the splint for 2 h/d, as well as being permitted to rest for 10 to 15 minutes after every 30 minutes of treatment. The splint could not be used for weight-bearing: only for nonweight-bearing positions. For hemiplegic children, the unaffected foot was not restricted or constrained. The children's usual physical therapy plans and AFO continued to be used throughout the observation period. Follow-up visits were done and the orthoses were changed as the children grew. The follow-up time was 6 months and 12 months after treatment.

### 2.3 Modified Ashworth scale and pedobarometric evaluation

The MAS of the gastrocsoleus of the affected feet was measured in a sitting position at the first visit to gauge muscle resistance to passive movement.^[24]^

A pedobarometric device was used to evaluate the plantar pressure of all subjects. The pedobarometric equipment consisted of a force plate (Gaitview AFA-50 system; alFOOTs, Seoul, Republic of Korea), validity and reliability of which had already been proven.^[25]^ The force plate has a 410 × 410 × 3 mm active area consisting of a 45-mm thick floor mat (area 700 × 500 mm), comprising 2304 (48 × 48) force-sensitive resistor sensors with a data sampling frequency of 17 Hz. A static test was performed 3 times on each subject in standing position, with arms by their sides, looking at a fixed point, for 5 seconds. The tests were performed on all TD children and CP children (before and after 6-month and 12-month treatment periods) with AFO + stretching and static AFO treatments.

### 2.4 Data processing

Peak pressure readings were taken under the heel and forefoot and recorded as a heel/forefoot pressure ratio. A high value indicated increased pressure under the heel, characteristic of a calcaneus deformity, whereas a low value indicated reduced heel pressure, associated with an equinus deformity (heel/forefoot ration of <1).^[26–29]^ If the value was <0.4, severe equinus deformity was diagnosed. To the contrary, if the value was >2, this implied a calcaneus deformity.^[29]^

### 2.5 Statistical analysis

For the patients included in this study, both feet of the TD children and all affected feet of the CP children were analyzed. Statistical analyses were performed using SPSS for Windows version 19.0 (IBM Corp, Armonk, NY). All parameters were tested for normality based on skewness and kurtosis. Paired *t* test and independent-samples *T* tests were utilized when data was normally distributed. A Wilcoxon rank-sum test and Mann-Whitney test were utilized if the data was not normally distributed. A descriptive statistical analysis of the quantitative parameters of mean and standard deviation was performed. A linear mixed model analysis with nested terms (subject within group) was used to account for random effects. Independent-samples *T* tests were used for each study group. Pairwise comparisons between the affected sides of the treatment groups were undertaken. The level of statistical significance was set at *P* <.05. A sample size of 80 patients would require above 85% power to detect a significant difference between the 2 CP groups with a significance level (alpha) of .05.

## 3 Results

From 88 children, 155 feet were analyzed in this study, which included 60 feet from 30 TD children, 45 feet from 28 CP children with AFO + stretching treatment, and 50 feet from 30 CP children with static AFO treatment. During the study period, 22 CP children were not included in the follow-up examination. There are 17 families who voluntarily gave up conservative treatment or chose other treatments, such as surgeries; 5 children failed to be connected after the first visit. Table 1 shows the demographic characteristics of the study cohort: male-to-female ratio, age, body height, weight, and affected feet for the 3 groups (TD, AFO + stretching treatment, and static AFO treatment). There were 45 affected limbs in AFO + stretching group (13 for MAS I, 15 for MAS I+, 14 for MAS II, and 3 for MAS III), and 50 affected limbs in static AFO group (5 for MAS I, 17 for MAS I+, 20 for MAS II, and 8 for MAS III).

**Table 1:**
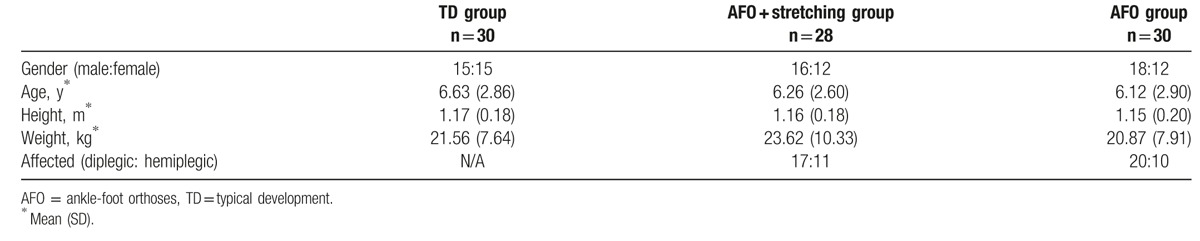
Demographic characteristics of the participants of this study.

**Figure 2:**
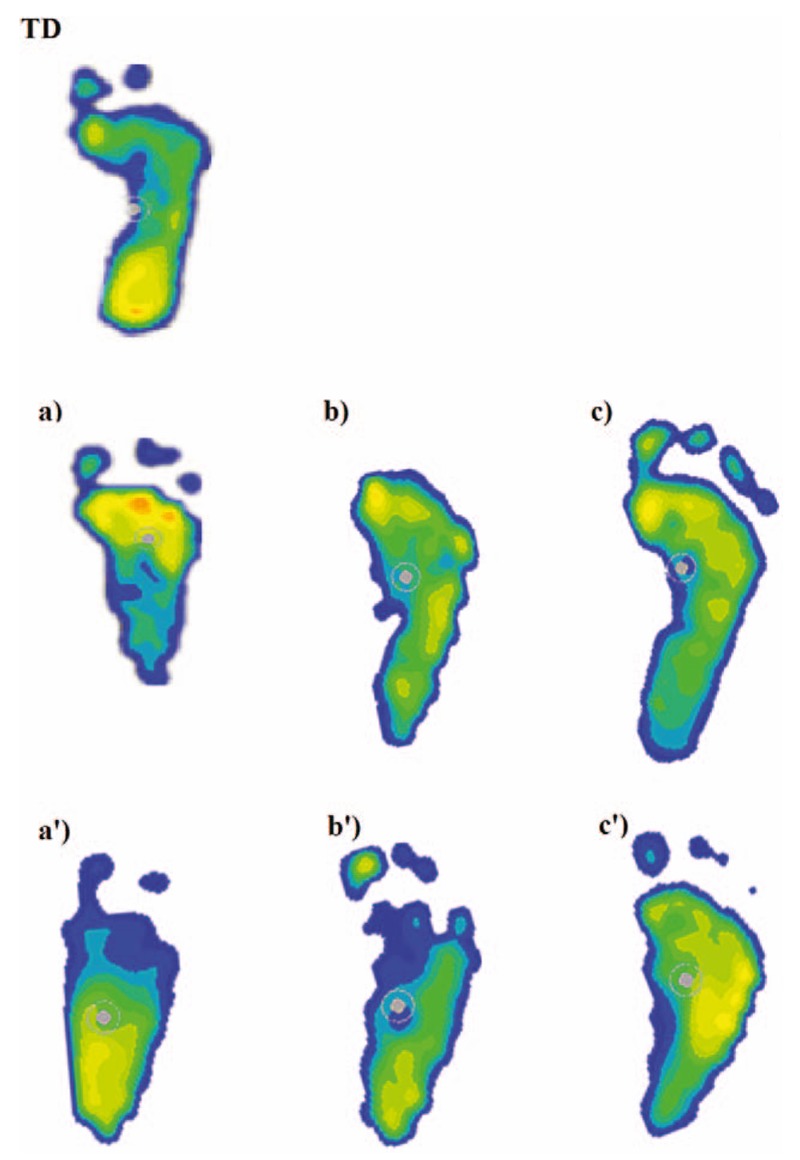
Typical footprints from each group—TD; AFO + stretching treatment group: (a) before, (b) 6 mo after treatment, and (c) 12 mo after treatment; and static AFO treatment group: (a′) before, (b′) 6 mo after treatment, and c′) 12 mo after treatment. AFO = ankle-foot orthoses, TD = typical development.

**Figure 3:**
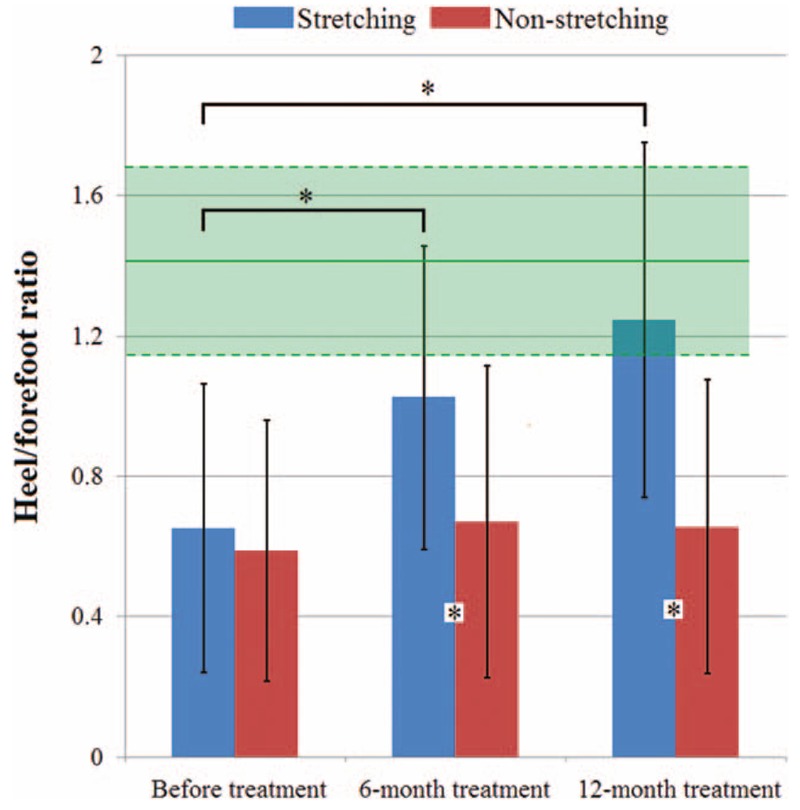
Changes in heel/forefoot ratio between AFO + stretching and AFO corrections before and after 6-mo and 12-mo treatments. AFO = ankle-foot orthoses.

Figure 2 shows a comparison of typical foot pressure distribution before and after 6-month and 12-month treatments using AFO + stretching (a–c) and static AFO (a′–c′). At follow-up examinations, the parents indicated that they were more satisfied with the AFO + stretching treatment. Figure 3 illustrates measurements of the heel/forefoot ratio for the 3 groups. The heel/forefoot ratio of the TD group was 1.41 ± 0.25 (green zone). The heel/forefoot ratios of the AFO + stretching treatment group before and after 6-month and 12-month treatments were, respectively, 0.65 ± 0.41, 1.02 ± 0.44, and 1.24 ± 0.51 (blue bars). The heel/forefoot ratios of the static AFO treatment group before and after 6-month and 12-month treatments were 0.59 ± 0.37, 0.67 ± 0.44, and 0.66 ± 0.42 (red bars). There was no significant difference in the heel/forefoot ratio between the AFO + stretching and static AFO treatment groups at the first visit (F1,93 = 1.44, *P* = .43). Significant differences in the heel/forefoot ratio were found between the before and after 6-month (*P* < .001) and 12-month (*P* < .001) follow-ups with AFO + stretching treatment. There were no significant differences in the heel/forefoot ratio between the before and after 6-month (*P* = .119) and 12-month (*P* = .151) follow-ups with static AFO treatment. The mean of the heel/forefoot ratio of the AFO + stretching group at the final visit was within the range of the TD measurements, which indicated that the AFO + stretching technique was effective for treating equinus.

An attempt was made to correlate the effectiveness of AFO + stretching treatment with patient age. However, after 6-month and 12-month treatments, changes in the heel/forefoot ratios showed a large dispersion across all ages (Table 2). The statistical results showed no correlation between treatment outcome of splint-assisted AFO correction and patient age (*P* = .326 and *P* = .398). However, a higher value of heel/forefoot ratio was recorded after 6 and 12 months than before treatment for each age group. This indicated that AFO + stretching would be effective for correcting equinus in the age groups tested. For the 10 to 12 years group, no significant difference was found in heel/forefoot ratio before and after treatment (*P* > .05).

**Table 2:**
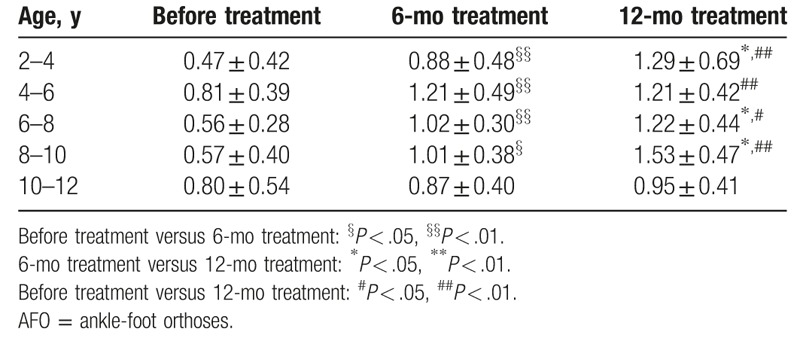
Heel/forefoot ratio of AFO + stretching group in different age groups.

**Figure 4:**
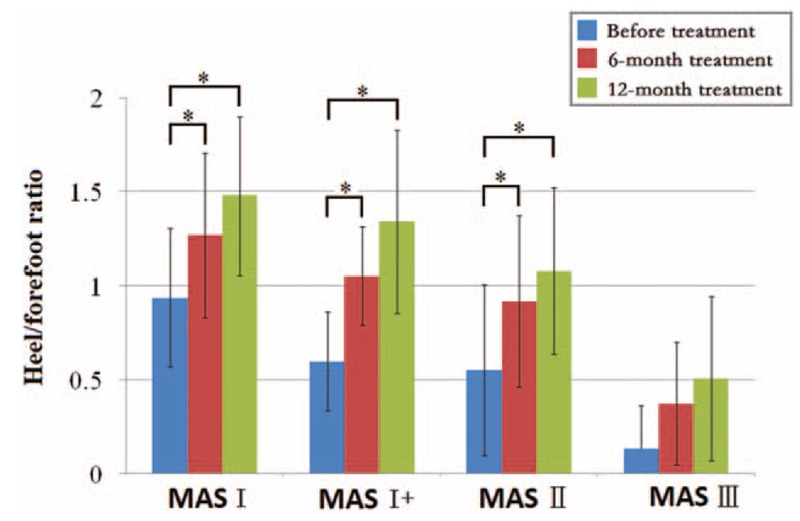
Heel/forefoot ratio before and after 6-mo and 12-mo AFO + stretching correction, with consideration for the MAS. AFO = ankle-foot orthoses, MAS = modified Ashworth scale.

Changes in the heel/forefoot ratios based on the MAS were compared to evaluate the effectiveness of the AFO + stretching treatment on the degree of muscle tone. Figure 4 illustrates that the heel/forefoot ratios gradually increased before and after the 6-month and 12-month treatments. The paired *T* test shows a significant improvement in MAS I, MAS I+, and MAS II groups after AFO + stretching treatment for both 6 months and 12 months (*P* = .039 and *P* = .006 for MAS I; *P* = .001 and *P* = .001 for MAS I+; *P* = .003 and *P* = .003 for MAS II). However, there was no difference in 6-month and 12-month treatments for MAS III (*P* = .593 and *P* = .285).

## 4 Discussion

AFO are considered to be an effective conservative treatment for preventing the progression of equinus deformities in children with CP. We developed a novel splint-assisted AFO to provide an adjustable sustained stretching for the treatment of equinus deformities of CP children. To accommodate individual differences in the severity of foot deformity, the splint was equipped with adjustable bars that were capable of providing a customizable stretching force for different stages of treatment. Each child was instructed to perform passive stretching for 2 h/d, with the splint adjusted to their individual tolerance; stretching the tissues close to the limit of the children's pain tolerance would be optimum for lengthening the muscles and tendons.^[30]^ As the contracture of the muscles and tendons relaxed, the equinus deformity could be more easily corrected with an AFO.

The design of the adjustable splint is based on the passive stretching for the contracture of muscles and tendons. Spasticity is the most common movement disorder in people with CP. Although spasticity is the result of the inhibition of motor neurons, the structure and mechanical properties of muscles and tendons also exhibit significant changes,^[31]^ such as shortening of the fibers and increased stiffness. Passive stretching is an important physical treatment for relaxing the contracture of muscles and tendons.^[5]^ Although the mechanism of contracture is not well understood, clinical evidence has shown that passive stretching could improve the range of movement and reduce spasticity in CP children.^[14]^ Passive stretching treatment can be divided into 2 categories: manual stretching and sustained stretching.^[14,32]^ Compared to manual stretching, sustained stretching is preferable to reduce equinus deformity and increase the range of motion. There is definitive evidence indicating that sustained stretching for no more than 30 minutes over a long-term period exhibits more effective outcomes when treating CP children than manual stretching.^[7]^

Based on these evidences,^[5]^ an adjustable splint-assisted AFO was developed to apply sustained stretching for treatment of CP children. To evaluate the outcome of treatment, the heel/forefoot ratio was introduced to represent the degree of equinus deformity, which is defined as the ratio of peak pressure under the heel to the maximum value under the forefoot.^[26]^ A lower value of the heel/forefoot ratio indicates a greater degree of equinus deformity. The ankle joint angle was also assessed. Compared to manual measurement of the joint angle, pedobarometric measurement is a more objective method of assessing functional deformities.^[26]^ The results in this study showed that the average heel/forefoot ratio for CP children reached normal levels after 12 months of splint-assisted AFO (Fig. 3). AFO treatment is considered to be effective for preventing further equinus deformity and helping to improve gait.^[9,10]^ However, the long-term clinical significance of AFO use is unclear.^[10,13,33]^ In this study, the long-term effects of treatment with an AFO were analyzed and no significant differences were found before and after treatment for 6 and 12 months (*P* = .119 and *P* = .151). A likely reason is that AFO are designed to hold the subtalar joint in a neutral position^[34]^ and the stretch strength of spastic muscles and tendons is not sufficient.

Patient age has been reported as a critical factor influencing the outcome of treatment for correcting foot deformities in CP children.^[9]^ This study presented changes in the heel/forefoot ratio of CP children 2 to 12 years old, after 6-month and 12-month treatments. Although the value of the heel/forefoot ratio increased at each successive follow-up in each age group, the statistical results showed no correlation between treatment outcome of splint-assisted AFO correction and children's age (*P* = .326 and *P* = .398). For the 10 to 12 age group, 3 of the 6 cases treated failed to be adequately corrected. This suggests that splint-assisted AFO correction could be more effective for younger CP children. The effectiveness of the splint-assisted AFO correction was also evaluated in terms of the severity of muscle tone. The statistical results show that the heel/forefoot ratio of the treated children with different MAS levels exhibited a step-by-step increase after 6-month and 12-month treatments, which suggests that AFO + stretching could be effective for correcting equinus in children with MAS below IV. However, the effectiveness of splint-assisted AFO correction is still unclear for CP children with MAS IV and MAS V.

From a biomechanical viewpoint, contractures in children with hypertonicity may cause a transformation of motor units resulting in shortening of muscle fibers, leading to further contracture.^[3,35]^ Increased movement may contribute to an increase in muscle belly length which, due to an adaptive response of the muscle, may involve an increase in the number of series sarcomeres.^[31]^ Therefore, stretching should have a positive effect on equinus. However, some studies have rightfully pointed out that sitting in an uncomfortable, stationary position for long periods is not particularly fun for children: as a result parents may be hesitant to use traditional stretching techniques.^[36]^ In the present study, the stretching splint could be used partly instead of manual stretching, and the stretching force could be adjusted according to the individual's pain tolerance. Also, wearing the stretching splint does not intervene in the child's typical daily activities, such as watching TV or doing homework. Therefore, most children would be able to withstand splint stretching treatments for longer periods.

In this paper, the splint provides sustained stretching to short muscles and tendons, improving the correction of equinus deformities. The treatment outcome was quantitatively evaluated using biomechanical methods. A heel/forefoot ratio was introduced to represent the degree of equinus deformity. An increase of the heel/forefoot ratio following treatment is offered as proof of the effectiveness of the proposed treatment for CP children. This quantitative description is not only objective but also reproducible, and could provide more useful parameters than single recurrence rates or clinical evaluation. However, this study has some limitations:

1. The corrective methods used do not consider severe foot deformities. Also, the stretching splint is used for controlling equinus, and may not be effective for controlling other types of deformity.
2. The bias of the patients recruited in the study is quite wide. There are many other conditions such as cooperation with physiotherapy and cognitive skills which may have an effect on the results.

## 5 Conclusion

This article introduced a novel manner for correcting equinus using an adjustable splint-assisted AFO for use on children with moderate CP. This method achieves more effective outcomes for controlling equinus, in comparison with conventional AFO-only treatment regimes. The results confidently indicate this as an appropriate treatment option for CP children with equinus deformities.

## Acknowledgments

The author would like to thank all participants in this study and the Rokab Pedorthic Center in Beijing for their help with recruiting patients. The author also acknowledges Colin Joseph McClean for his recommendations on the preparation of this manuscript.

## Supplementary Material

Supplemental Digital Content
