# Supplementary material for: Conservative treatment for equinus deformity in children with cerebral palsy using an adjustable splint-assisted ankle-foot orthosis
Source: Medicine (Baltimore). 2017 Oct 27;96(40):e8186. doi: 10.1097/MD.0000000000008186 (PMC5738005; doi:10.1097/MD.0000000000008186)
Supplement: Supplemental Digital Content [file medi-96-e8186-s001.pdf]

北京航空航天大学生物与医学工程学院涉及人的生物医学研究伦理审查证明

编号(No): **20120205**

申请日期: 2012年 2月 5日

项目名称：痉挛型脑瘫足踝的生物力学评价

项目负责人：　樊瑜波

职称：　教授（博导）

电话：　86-010-82339861　　　　　　　　邮箱：　yubofan@buaa.edu.cn

研究者：陈薇	职称：　博士生	研究者：蒲放	职称：教授（博导）
研究者：杨阳	职称：　博士生	研究者：刘笑宇	职称：博士
研究者：王丽珍	职称：博士（讲师）	研究者：刘宏	职称：主治医

研究内容：本项目将研究基于生物力学的矫正方式，对矫正力的施加部位，施加方向，作用的大小以及作用结果进行研究。通过建立一种基于足部三维几何特征、表面变形、步态特征测量对外载作用下足部的生物力学响应进行评价的方法，将生物力学特征运用于儿童足部畸形评价，用于描述儿童足部畸形状态，并预测其发展情况，从而为选择适当的治疗方案，以及优化矫形器设计提供重要依据。

保密要点：<br>将对参与者的隐私严格保密，所有数据将以研究编号数字而非姓名加以标识。可以识别身份的信息将不会透露给研究小组以外的成员，除非获得参与者的许可。所有的研究成员和研究申办方都被要求对参与者身份保密。参与者的档案将保存在有锁的档案柜中，仅供研究人员审阅。

审查要点：	1. 研究的设计和实施是否科学，可行？<br>2. 受试者的医疗和保护<br>3. 受试者隐私的保护<br>4. 知情同意的过程<br>5. 其他

审查结果：	生物与医学工程伦理委员会意见	☑ 同意	☐ 不同意	☐ 修改

伦理委员会主任委员签章：

伦理委员会签章：

填表说明：1. 申请日期请填写拟交申请日期，编号由生物与医学工程伦理委员会填写。

2. 申请书中方格可在文字输入打印后，在选中的项目前用笔画√。

3. 联系人为：本研究项目的联系人及电话。

4. 研究者包括合作研究单位的人员。
